# Nationwide study of trends in physician partner choice for childbearing unions

**DOI:** 10.1111/joim.13464

**Published:** 2022-03-02

**Authors:** Linus Andersson, Margarita Chudnovskaya, Roujman Shahbazian, Cyrus Ghaznavi, Peter Ueda

**Affiliations:** ^1^ Swedish Institute for Social Research Stockholm University Stockholm Sweden; ^2^ Medical Education Program Washington University School of Medicine St. Louis Missouri USA; ^3^ Clinical Epidemiology Division Department of Medicine Karolinska Institutet Solna Stockholm Sweden

Dear Editor,

For physicians who wish to have children, their choice of partner might impact work hours [1, 2], career decisions [1, 2, 3], time spent caring for children [2] and relationship satisfaction [4]. As such, purported increases in *dual physician households* [2, 3] or *medical marriages* [4] (physicians who partner with other physicians; a phenomenon called *endogamy* [5]) have been subject to much attention. However, data on physicians’ partners from population‐based samples are scarce and trends over time in physician partner choice have not been assessed.

We used nationwide administrative registers in Sweden (described in Supporting Information Appendix), including the total population register and educational registers. We included physicians, born between 1941 and 1975, who had completed their medical education or had their medical qualifications registered in Sweden by age 42. We identified all individuals with whom the physicians had at least one child through age 42, based on all childbearing events, including biological (live births) or adopted children. We categorized physicians into 5‐year birth cohorts and described the 10 most common educational fields (focus of educational program) [6] of physicians’ partners, using categories described in Table S1. Analyses were performed by sex using Stata version 16.1 (StataCorp). The Regional Ethics Committee in Stockholm, Sweden, approved the study.

Analyses included 16,385 female physicians and their 14,024 partners and 21,300 male physicians and their 18,415 partners. The number of male physicians varied by birth cohort with no apparent trend over time. The number of female physicians had increased across birth cohorts (Table S2).

Figure 1 shows the 10 most common educational fields of partners to physicians born in 1941–1945, 1956–1960 and 1971–1975 (Tables S3 and S4 show data for all birth cohorts). Female physicians most commonly partnered with physicians (22.9% of partners) and engineers (15.7%). This was consistent across birth cohorts, although the proportion who were physicians had increased slightly from 19.9% to 25.9% among partners to female physicians born between 1941 and 1945 versus 1971 and 1975. Male physicians most commonly partnered with physicians (18.4%), nurses (18.1%) and teachers (15.3%). When comparing partners to male physicians born between 1941and 1945 versus 1971 and 1975, the proportion who were teachers had decreased from 22.6% to 9.3%, and the proportion who were nurses had decreased from 19.3% to 11.8% while the proportion who were physicians had more than doubled, from 12.0% to 29.8%.

**Fig. 1:**
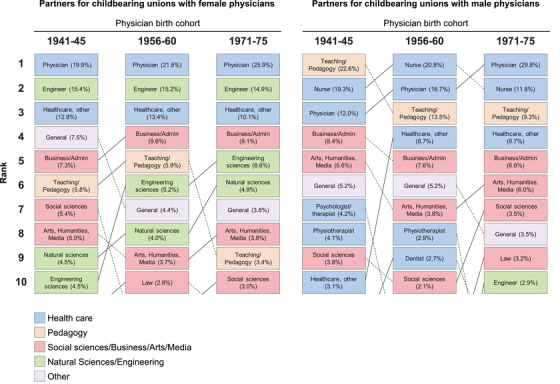
The most common educational fields of partners in childbearing unions with physicians born in 1941–1945, 1956–1960 and 1971–1975.

Female physicians in Sweden born between 1941 and 1975 most commonly partnered with engineers and physicians for their childbearing unions; this tendency remained consistent across birth cohorts, although the proportion of partners who were physicians had increased slightly. Male physicians born in 1941–1960 most commonly partnered with teachers or nurses; this later changed to physicians among those born in 1961–1975, with this coinciding with an increased number of female physicians in the country. Of the partners to both female and male physicians born in 1971–1975, nearly 30% were physicians. While the purported increase in “dual physician households” has received much attention in recent medical literature [2, 3], such an increase had occurred predominantly among male physicians in our study; for female physicians, partnering with another physician had been common for decades.

While our study is limited to physicians in Sweden, our findings align with studies from various populations and birth cohorts showing that highly educated women in heterosexual relationships, on the group level, tend to partner with men of equal or higher education or income, with this inclination being attributed to both preference and availability [7]. In contrast, partner choice of highly educated men tends to vary more significantly [7, 8, 9]. Moreover, in accordance with our findings, analyses of 25‐ to 50‐year‐old physicians in a US nationally representative survey (2000–2015) [1] showed that 31.4% of married female physicians and 17.1% of married male physicians had partnered with physicians. Our analyses used nationwide register data (rather than survey data) with nearly complete coverage with respect to educational characteristics and partners for childbearing unions, also included unmarried physicians, assessed partners with whom physicians had children (rather than marriage), examined changes over time and provided data also on partners who were not healthcare professionals.

Our analyses only accounted for childbearing unions that were captured in the administrative registers: registration of same sex childbearing unions started in 2005. To ensure comparability across birth cohorts, we did not include childbearing events occurring after age 42; however, vital statistics show that almost all women and around 94% of men who had children did so before age 40 [10].

Partner occupation (and income) could affect decisions that influence career progression [1, 2, 3] and work–life balance such as within‐couple distribution of parental leave and childbearing responsibilities [2]. The convergence of male and female physicians’ partner choice and its implications for physicians’ personal and professional lives merit further examination.

## Conflict of interest

The authors declare no conflict of interest.

## Author contributions

Linus Andersson had full access to all the data in the study and take responsibility for the integrity of the data and the accuracy of the data analysis. Concept and design: Linus Andersson, Margarita Chudnovskaya and Peter Ueda. Acquisition, analysis, or interpretation of data, and critical revision of the manuscript for important intellectual content: All authors. Drafting of the manuscript: Linus Andersson, Margarita Chudnovskaya, Cyrus Ghaznavi and Peter Ueda. Statistical analysis: Linus Andersson and Peter Ueda.

## Funding information

None.

## Supporting information

Online Appendix
