# Supplementary material for: Nationwide study of trends in physician partner choice for childbearing unions
Source: J Intern Med. 2022 Mar 2;292(1):165–7. doi: 10.1111/joim.13464 (PMC9314936; doi:10.1111/joim.13464)
Supplement: Supplementary file 1 — Online Appendix [file JOIM-292-165-s001.docx]

**Online Appendix**

Andersson L et al. Nationwide study of trends in physician partner choice for childbearing unions.

**Data sources**

The Swedish Total Population Register contains data on the vital status, date of birth and emigration/immigration of all individuals domiciled in Sweden at any time since the census of 1960.[1] The multigenerational register links each individual to their parents and thus enable linkage of individuals to their reproductive partners through their common biological or adopted child.[2] If a mother is married or recently widowed at the time of the child’s birth the husband is registered as the father. In other scenarios, paternity is determined by acknowledgement, or, more rarely, by a court.

As same-sex partnerships for childbearing were not registered before 2005 and such partnerships remain rare our analyses primarily represent childbearing heterosexual partnerships.

The educational register is based on information obtained from the population censuses of 1985 and 1990, and annual reports regarding individuals’ completion of courses, enrollment and obtained degrees in the entire Swedish educational system.[3,4] For individuals who immigrated to Sweden, Statistics Sweden has complemented the data by conducting large-scale annual mail inquiries. Education is coded based on the SUN (Swedish Standard Classification of Education) classification,[5] which categorizes educational programs and courses based on level (e.g. shorter university degree, master, or research degree) and educational field (e.g. nursing, law, engineering). We analyzed educational field as described in eTable 1. Categorization of educational fields were based on previous literature[4] and modified in order to identify specific occupations (e.g. nurse, physiotherapist, engineer) or educational areas (e.g. social sciences, business and administration, law) for educational programs that do not lead to specific occupations.

**eTable 1** Categorization of educational field of physicians’ partners for childbearing unions according to SUN2020 codes.

| **Educational field category** | **SUN2020 codes** |
| --- | --- |
| Physician | 721a, 721b, 721c, 721d, 721e, 721f, 721g, 721h, 721i, 721j, 721k, 721l, 721m |
| Dentist | 724a, 724b, 724c |
| Psychologist/psychotherapist | 311a, 311b, 311c |
| Medical physicist | 725e |
| Pharmacist/Prescriptionist/Other pharmaceutical | 727a, 727b, 727x |
| Speech therapist | 726c |
| Chiropractor/Naprapath/Other rehabilitation | 726e, 726f, 726x |
| Law | 38 |
| Engineer (including architect) | 520a, 520b, 521a, 521b, 522a, 522b, 523a, 523b, 523c, 523d, 524a, 524b, 525a, 525b, 526a, 526b, 527a, 527b, 529a, 529b, 540a, 540b, 541a, 541b, 542a, 542b, 543a, 543b, 544a, 544b, 580a, 580b, 581a, 581b, 581c, 581d, 581e, 582a, 582b |
| Biomedical scientist | 725d |
| Nurse | 723a, 723b, 723c, 723d, 723e, 723f, 723g, 723h, 723i, 723j, 723k, 723l, 723m, 723n, 725f, 724e |
| Teaching/pedagogy | 1 |
| Physiotherapist/occupational therapist | 726a, 726b |
| Optician/audiologist | 725a, 725b |
| Dietician | 726d |
| Agriculture/animal care | 6 |
| Dental care (excl dentist) | 724d, 724f, 724x |
| Natural sciences | 42, 44, 46 |
| Social Sciences | 310a, 310b, 310x, 311x, 312a, 312b, 312c, 312x, 313z, 314a, 314b, 314x, 319z |
| Business/administration/trade | 34 |
| Engineering sciences (excl engineers) | 520x, 521c, 521x, 522c, 522d, 522x, 523e, 523f, 523x, 524c, 524d, 524x, 525c, 525d, 525x, 526x, 527x, 581x, 529x, 540x, 541c, 541d, 541e, 541x, 542c, 542d, 542x, 543c, 543d, 543e, 543f, 543x, 544c, 544x, 549z, 580x |
| Arts/Humanities/Media/Religion | 2, 320, 321, 322, 329 |
| Law enforcement/Security/Military | 86 |
| Orthopaedic engineer/technician | 725c |
| IT | 48 |
| Healthcare, other | 720z, 721x, 725x, 729z, 762e |
| Caregiving, healthcare/service homes | 723o, 723p, 723q, 723r, 723s, 723t, 723u, 723x |
| Construction work | 582c, 582d, 582e, 582f, 582g, 582h, 582i, 582x, 589z |
| Personal services/Travel/Tourism/Cleaning | 81, 85 |
| Transport | 84 |
| General | 0 |

Categorization of educational fields were based on previous literature[4] and modified in order to identify educational programs that correspond to specific occupations (e.g. nurse, physiotherapist, engineer) or educational areas (e.g. social sciences, business and administration, law) for educational programs that do not lead to specified occupations.

For individuals with completed educational programs or courses from more than one category a hierarchical categorization was performed based on the order in which the categories are presented in the table. The hierarchy was determined based on educational level, with priority given to higher educational levels, and educational field, with priority given to educations/occupations within health care.

Categories are presented as SUN2020 codes, which are largely similar to those based on SUN2000, the previous coding system for classification of education in Sweden. [5]

**eTable 2** Number of female and male physicians and their partners for childbearing unions by physician birth cohort.

| Physician birth cohort | Female physicians* | Partners to female physicians | Male physicians* | Partners to male physicians |
| --- | --- | --- | --- | --- |
| 1941-45 | 1306 | 1109 | 2189 | 2016 |
| 1946-50 | 1981 | 1748 | 3407 | 3171 |
| 1951-55 | 2287 | 1935 | 3413 | 2987 |
| 1956-60 | 2413 | 2031 | 3187 | 2715 |
| 1961-65 | 2298 | 1928 | 2923 | 2386 |
| 1966-70 | 2666 | 2278 | 2828 | 2337 |
| 1971-75 | 3434 | 2995 | 3353 | 2803 |
| Total | 16385 | 14024 | 21300 | 18415 |

*Including physicians who were not part of any childbearing union.

**eTable 3** Ten most common educational fields of partners for childbearing unions with female physicians by physician birth cohort.

| **Rank** | **1941-45** | N (%) | **1946-50** | N (%) | **1951-55** | N (%) | **1956-60** | N (%) |
| --- | --- | --- | --- | --- | --- | --- | --- | --- |
| 1 | Physician | 199 (19.9) | Physician | 349 (21.8) | Physician | 388 (21.6) | Physician | 403 (21.8) |
| 2 | Engineer | 154 (15.4) | Engineer | 229 (14.3) | Engineer | 274 (15.2) | Engineer | 280 (15.2) |
| 3 | Healthcare, other | 128 (12.8) | Healthcare, other | 208 (13) | Healthcare, other | 266 (14.8) | Healthcare, other | 247 (13.4) |
| 4 | General | 75 (7.5) | Teaching/pedagogy | 112 (7) | Business/administration | 135 (7.5) | Business/administration | 178 (9.6) |
| 5 | Business/administration | 73 (7.3) | General | 97 (6.1) | General | 106 (5.9) | Teaching/pedagogy | 109 (5.9) |
| 6 | Teaching/pedagogy | 58 (5.8) | Business/administration | 95 (5.9) | Teaching/pedagogy | 103 (5.7) | Engineering sciences | 96 (5.2) |
| 7 | Social Sciences | 54 (5.4) | Social Sciences | 82 (5.1) | Engineering sciences | 87 (4.8) | General | 82 (4.4) |
| 8 | Arts/Humanities/Media | 50 (5) | Engineering sciences | 80 (5) | Social Sciences | 69 (3.8) | Natural sciences | 74 (4) |
| 9 | Engineering sciences | 45 (4.5) | Arts/Humanities/Media | 72 (4.5) | Natural sciences | 64 (3.6) | Arts/Humanities/Media | 69 (3.7) |
| 10 | Natural sciences | 45 (4.5) | Natural sciences | 70 (4.4) | Arts/Humanities/Media | 55 (3.1) | Law | 51 (2.8) |
|  |  |  |  |  |  |  |  |  |
| **Rank** | **1961-65** | N (%) | **1966-70** | N (%) | **1971-75** | N (%) | **Total** | N (%) |
| 1 | Physician | 395 (22.6) | Physician | 497 (23.8) | Physician | 724 (25.9) | Physician | 2955 (22.9) |
| 2 | Engineer | 332 (19) | Engineer | 334 (16) | Engineer | 417 (14.9) | Engineer | 2020 (15.7) |
| 3 | Healthcare, other | 254 (14.5) | Healthcare, other | 220 (10.5) | Healthcare, other | 283 (10.1) | Healthcare, other | 1606 (12.5) |
| 4 | Business/administration | 138 (7.9) | Business/administration | 195 (9.3) | Business/administration | 255 (9.1) | Business/administration | 1069 (8.3) |
| 5 | Engineering sciences | 115 (6.6) | Engineering sciences | 149 (7.1) | Engineering sciences | 241 (8.6) | Engineering sciences | 813 (6.3) |
| 6 | General | 84 (4.8) | General | 89 (4.3) | Natural sciences | 136 (4.9) | General | 640 (5) |
| 7 | Teaching/pedagogy | 66 (3.8) | Teaching/pedagogy | 87 (4.2) | General | 107 (3.8) | Teaching/pedagogy | 629 (4.9) |
| 8 | Natural sciences | 62 (3.5) | Natural sciences | 78 (3.7) | Arts/Humanities/Media | 105 (3.8) | Natural sciences | 529 (4.1) |
| 9 | Arts/Humanities/Media | 55 (3.1) | Arts/Humanities/Media | 76 (3.6) | Teaching/pedagogy | 94 (3.4) | Arts/Humanities/Media | 482 (3.7) |
| 10 | Law | 45 (2.6) | Social Sciences | 54 (2.6) | Social Sciences | 84 (3) | Social Sciences | 415 (3.2) |

n= 1145 of the partners had unknown education and were not included in the denominator.

**eTable 4** Ten most common educational fields of partners for childbearing unions with male physicians by physician birth cohort.

| **Rank** | **1941-45** | **N (%)** | **1946-50** | **N (%)** | **1951-55** | **N (%)** | **1956-60** | **N (%)** |
| --- | --- | --- | --- | --- | --- | --- | --- | --- |
| 1 | Teaching/pedagogy | 415 (22.6) | Teaching/pedagogy | 644 (22.1) | Nurse | 675 (24.2) | Nurse | 529 (20.8) |
| 2 | Nurse | 354 (19.3) | Nurse | 620 (21.3) | Teaching/pedagogy | 454 (16.3) | Physician | 426 (16.7) |
| 3 | Physician | 221 (12) | Physician | 353 (12.1) | Physician | 408 (14.6) | Teaching/pedagogy | 344 (13.5) |
| 4 | Business/administration | 155 (8.4) | Business/administration | 191 (6.6) | Business/administration | 183 (6.6) | Healthcare, other | 221 (8.7) |
| 5 | Arts/Humanities/Media | 103 (5.6) | General | 164 (5.6) | Healthcare, other | 183 (6.6) | Business/administration | 194 (7.6) |
| 6 | General | 95 (5.2) | Arts/Humanities/Media | 129 (4.4) | General | 123 (4.4) | General | 132 (5.2) |
| 7 | Psychologist/psychotherapist | 77 (4.2) | Healthcare, other | 116 (4) | Arts/Humanities/Media | 98 (3.5) | Arts/Humanities/Media | 98 (3.8) |
| 8 | Physiotherapist/occupational therapist | 76 (4.1) | Social Sciences | 104 (3.6) | Physiotherapist/occupational therapist | 93 (3.3) | Physiotherapist/occupational therapist | 74 (2.9) |
| 9 | Social Sciences | 69 (3.8) | Psychologist/psychotherapist | 103 (3.5) | Social Sciences | 80 (2.9) | Dentist | 69 (2.7) |
| 10 | Healthcare, other | 57 (3.1) | Physiotherapist/occupational therapist | 103 (3.5) | Psychologist/psychotherapist | 76 (2.7) | Social Sciences | 54 (2.1) |
|  |  |  |  |  |  |  |  |  |
| **Rank** | **1961-65** | **N (%)** | **1966-70** | **N (%)** | **1971-75** | **N (%)** | **Total** | **N (%)** |
| 1 | Physician | 441 (19.3) | Physician | 539 (23.9) | Physician | 810 (29.8) | Physician | 3198 (18.4) |
| 2 | Nurse | 336 (14.7) | Nurse | 313 (13.9) | Nurse | 321 (11.8) | Nurse | 3148 (18.1) |
| 3 | Teaching/pedagogy | 295 (12.9) | Teaching/pedagogy | 256 (11.3) | Teaching/pedagogy | 254 (9.3) | Teaching/pedagogy | 2662 (15.3) |
| 4 | Healthcare, other | 216 (9.5) | Business/administration | 203 (9) | Healthcare, other | 237 (8.7) | Business/administration | 1282 (7.4) |
| 5 | Business/administration | 177 (7.8) | Healthcare, other | 192 (8.5) | Business/administration | 179 (6.6) | Healthcare, other | 1222 (7) |
| 6 | Arts/Humanities/Media | 107 (4.7) | Arts/Humanities/Media | 133 (5.9) | Arts/Humanities/Media | 164 (6) | Arts/Humanities/Media | 832 (4.8) |
| 7 | General | 102 (4.5) | Engineer | 70 (3.1) | Social Sciences | 95 (3.5) | General | 776 (4.5) |
| 8 | Social Sciences | 72 (3.2) | Social Sciences | 69 (3.1) | General | 94 (3.5) | Social Sciences | 543 (3.1) |
| 9 | Law | 54 (2.4) | General | 66 (2.9) | Law | 88 (3.2) | Physiotherapist/occupational therapist | 469 (2.7) |
| 10 | Natural sciences | 54 (2.4) | Natural sciences | 59 (2.6) | Engineer | 79 (2.9) | Psychologist/psychotherapist | 406 (2.3) |

n= 1069 of the partners had unknown education and were not included in the denominator.

**References**

1 Ludvigsson JF, Almqvist C, Bonamy A-KE, *et al.* Registers of the Swedish total population and their use in medical research. *Eur J Epidemiol* 2016;**31**:125–36. doi:10.1007/s10654-016-0117-y

2 Ekbom A. The Swedish Multi-generation Register. In: *Methods in molecular biology (Clifton, N.J.)*. Methods Mol Biol 2011. 215–20. doi:10.1007/978-1-59745-423-0_10

3 Statistics Sweden. Background facts - Labour and Education statistics. 2019.https://www.scb.se/contentassets/f0bc88c852364b6ea5c1654a0cc90234/lisa-bakgrundsfakta-1990-2017.pdf (accessed 20 Jul 2020).

4 Hoem JM, Neyer G, Andersson G. Education and childlessness: The relationship between educational field, educational level, and childlessness among Swedish women born in 1955-59. *Demogr Res* 2006;**14**:331–80. doi:10.4054/DemRes.2006.14.15

5 Statistics Sweden. SUN 2020 - Swedish Standard Classification of Education. https://www.scb.se/contentassets/aeeedec0e28c465aa524429407dcd5ba/sun-2020_version-1.1.pdf (accessed 22 Jul 2020).
